# Supplementary material for: Beta Human Papillomavirus 8E6 Attenuates Non-Homologous End Joining by Hindering DNA-PKcs Activity
Source: Cancers (Basel). 2020 Aug 20;12(9):2356. doi: 10.3390/cancers12092356 (PMC7564021; doi:10.3390/cancers12092356)
Supplement: Supplementary file 1 [file cancers-12-02356-s001.pdf]

Article

Beta Human Papillomavirus 8E6 Attenuates Non-Homologous End Joining by Hindering DNA-PKcs Activity

Changkun Hu, Taylor Bugbee, Monica Gamez and Nicholas A. Wallace

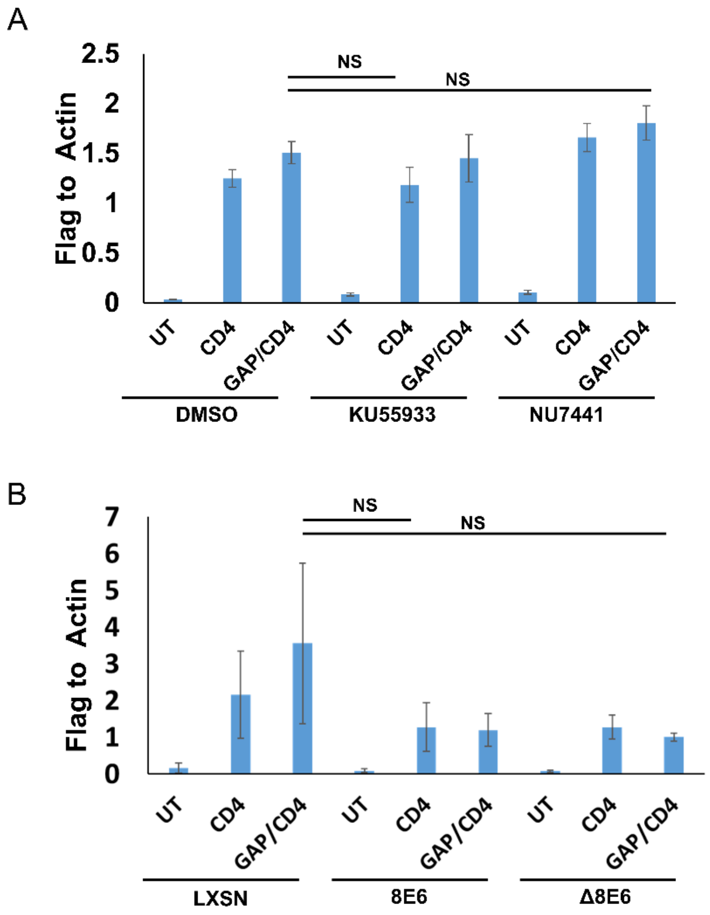

Figure S1. Transfection efficiency represented by FLAG tagged SgRNA-CAS9 targeting CD4 and GAPDH. NS: Not significant.

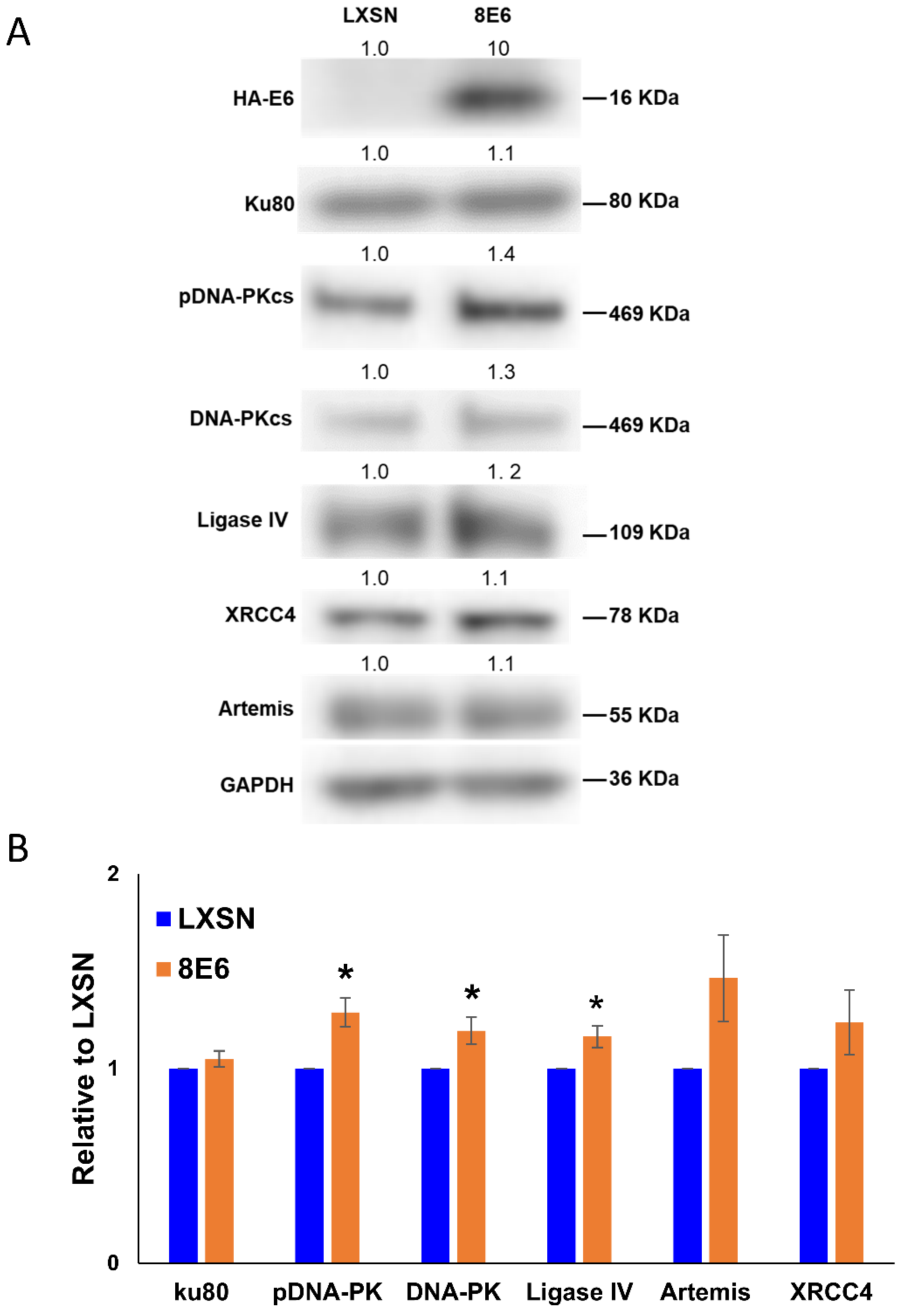

Figure S2. β-HPV 8 E6 does not decrease NHEJ protein in untreated cells. * indicates $p < 0.05$.

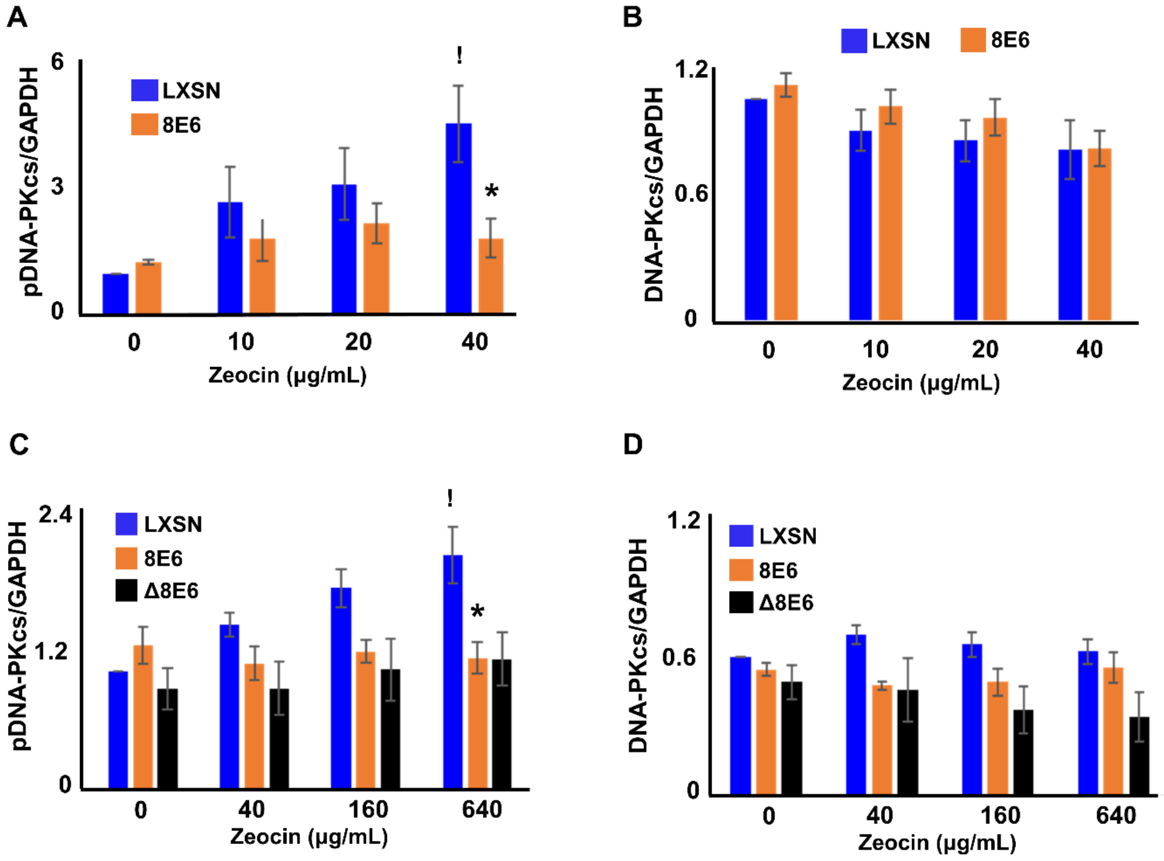

Figure S3. pDNA-PKcs and total DNA-PKcs normalized to GAPDH in HFK and U2OS. (**A**) HFK cells. (**B**) HFK cells. (**C**) U2OS cells. (**D**) U2OS cells. * indicates $p < 0.05$. ! indicates significant difference between Zeocin treated and untreated group.

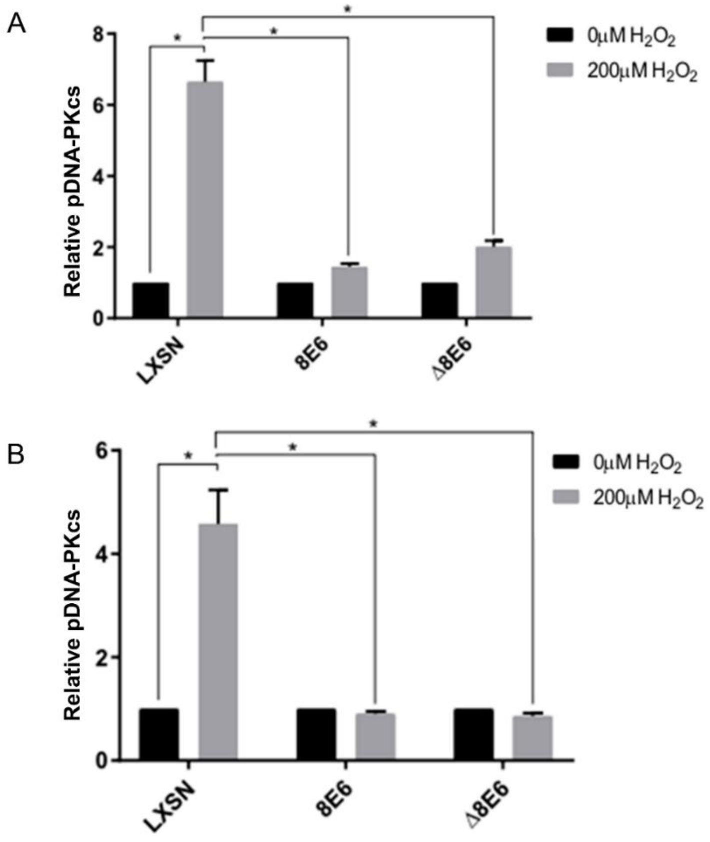

Figure S4. β-HPV 8 E6 decreases H_2O_2 induced DNA-PKcs phosphorylation. * indicates $p < 0.05$.

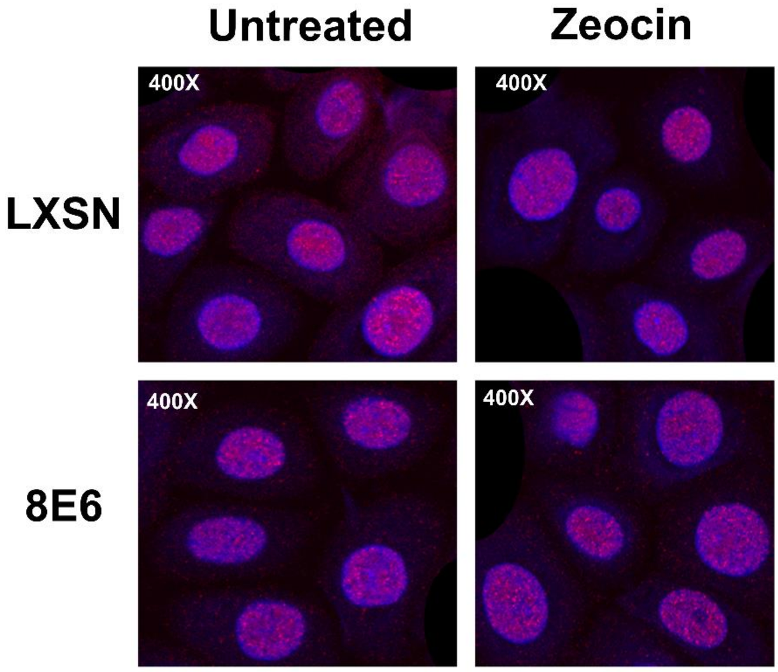

Figure S5. Total DNA-PKcs shows pan-nuclear expression in HFK cells. All microscopy images are 400X magnification.

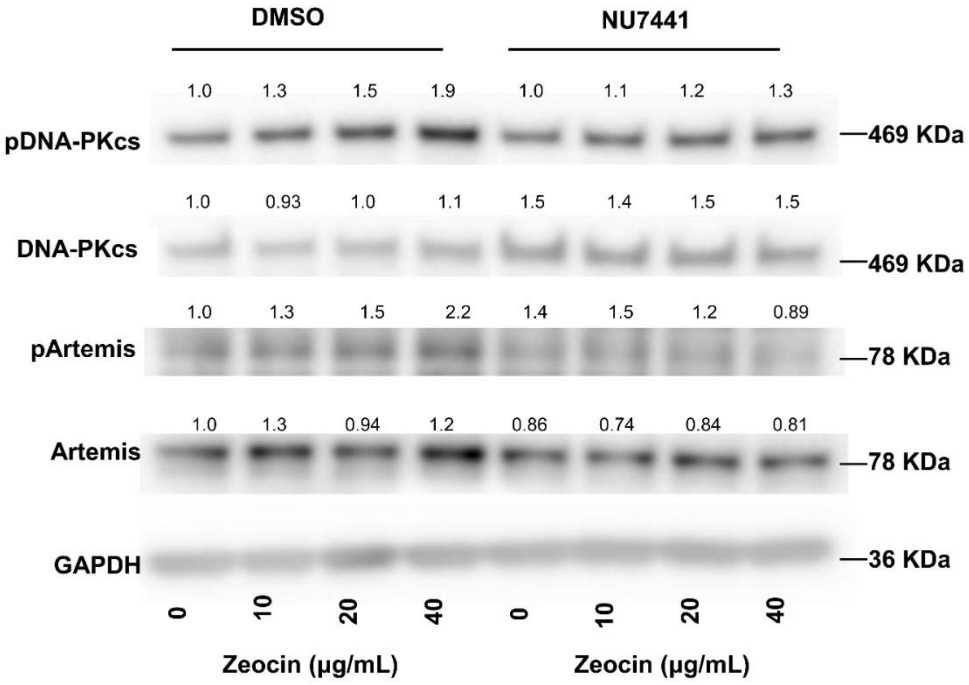

Figure S6. Inhibiting DNA-PK decreases Artemis phosphorylation.

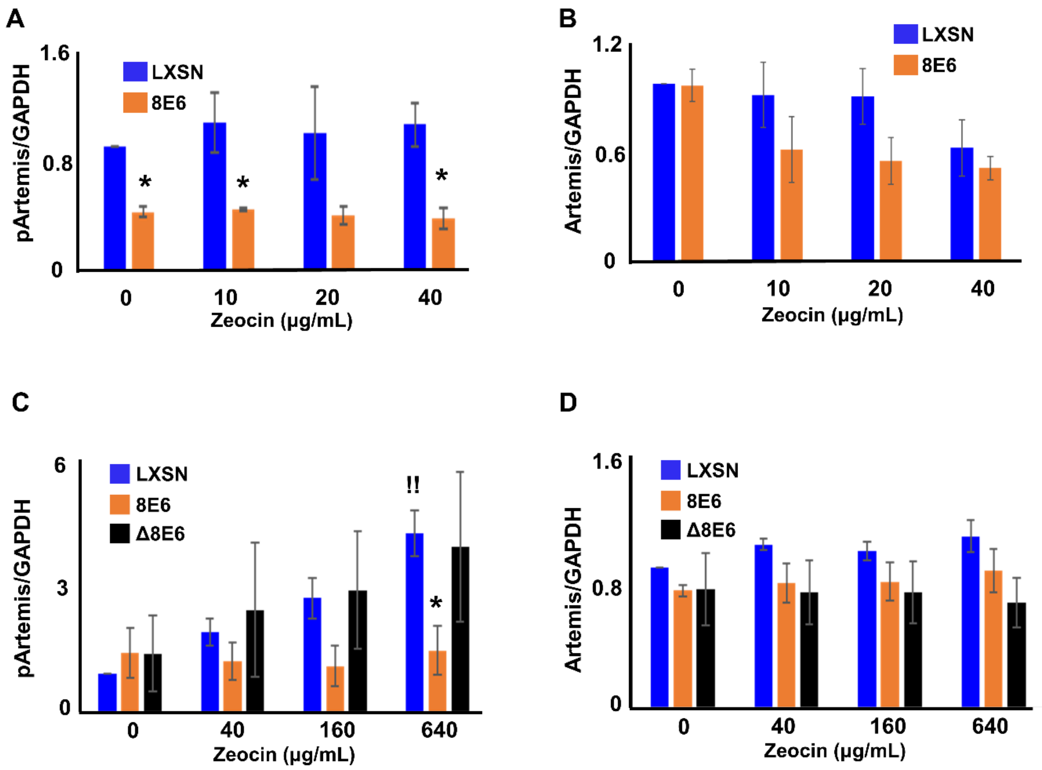

Figure S7. pArtemis and total Artemis normalized to GAPDH in HFK and U2OS cells. (**A**) HFK cells. (**B**) HFK cells. (**C**) U2OS cells. (**D**) U2OS cells. * indicates $p < 0.05$. !! indicates significant difference between Zeocin treated and untreated group (p<0.01).

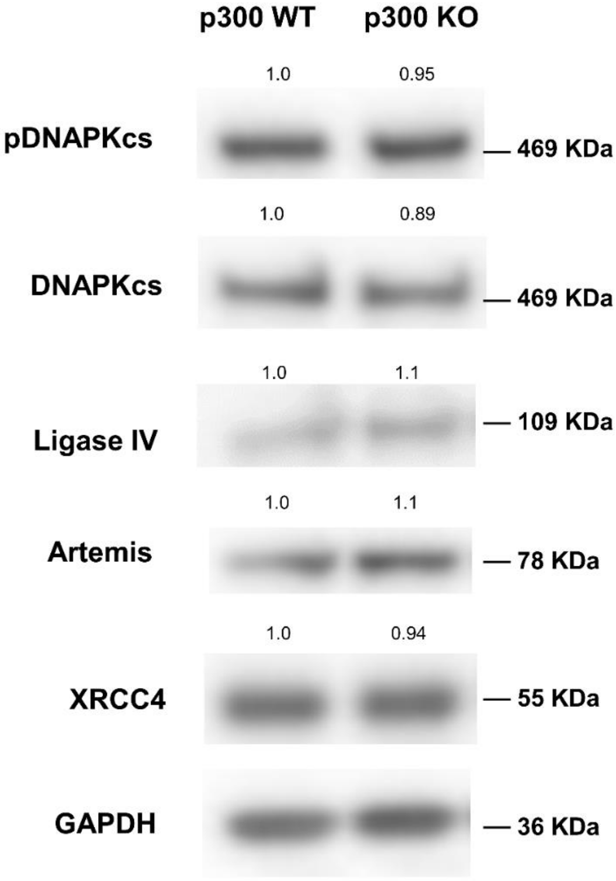

Figure S8. p300 knockout does not decrease major NHEJ proteins in untreated cells.

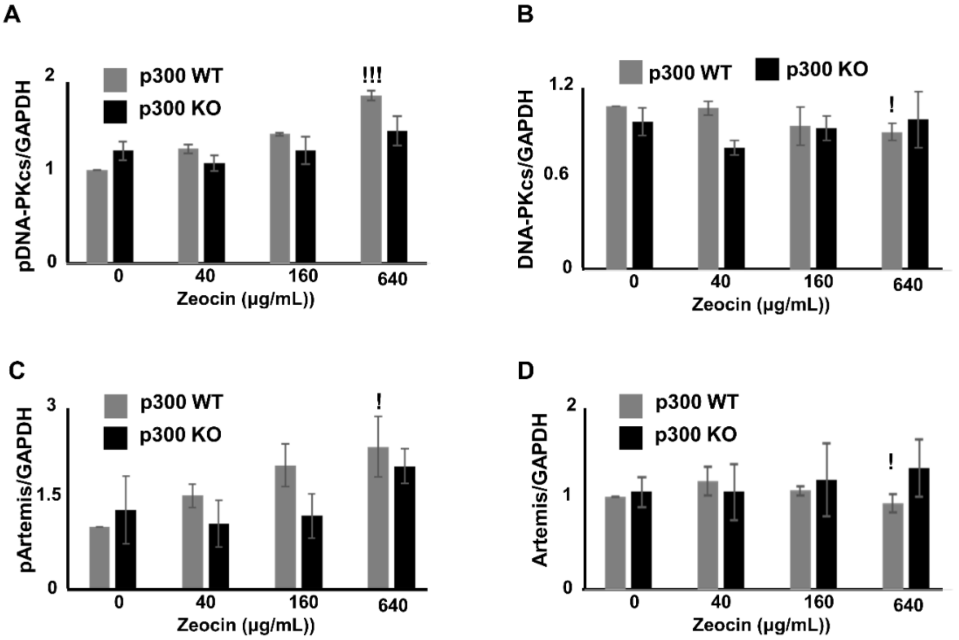

Figure S9. pDNA-PKcs, total DNA-PKcs, pArtemis, and total Artemis normalized to GAPDH in HCT cells. ! indicates significant difference between Zeocin treated and untreated group. !!! indicates significant difference between Zeocin treated and untreated group (p<0.001).

Figure 1B

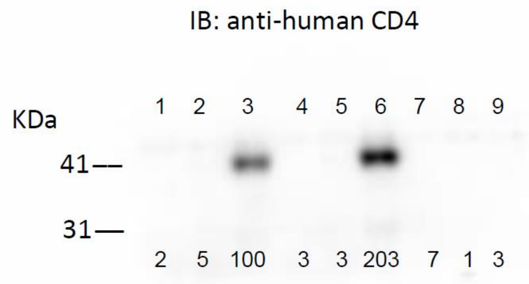

Figure 1B

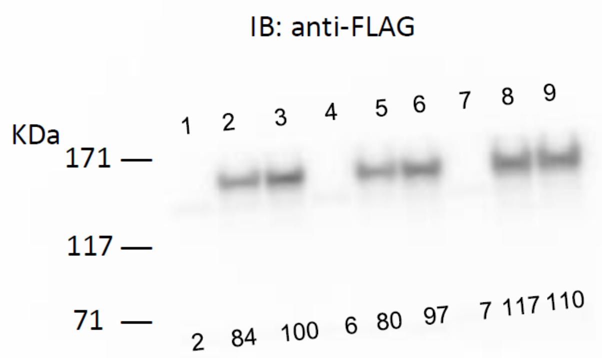

Figure 1B

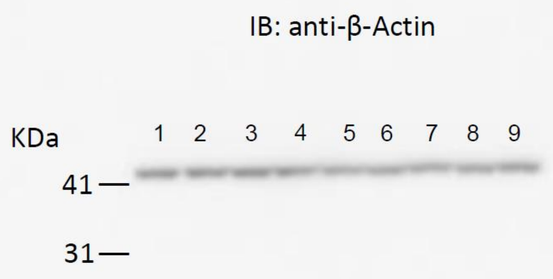

Figure 1D

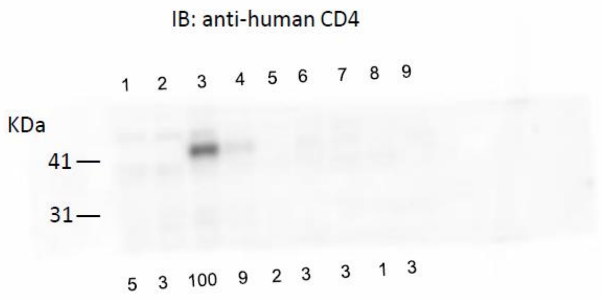

Figure 1D

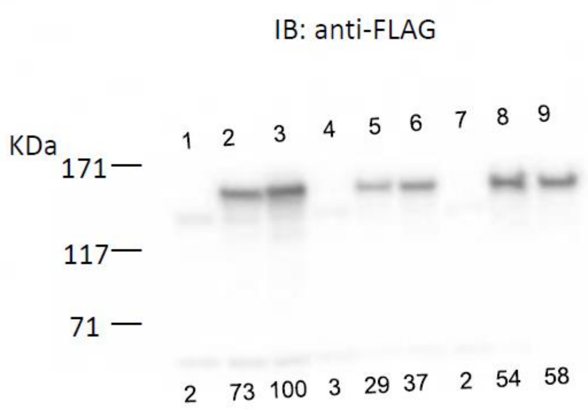

Figure 1D

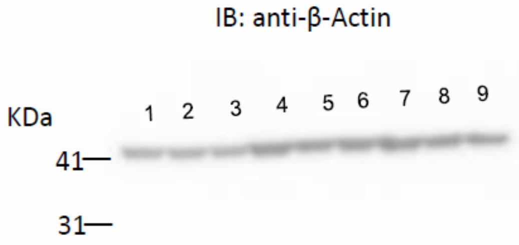

Figure 1F

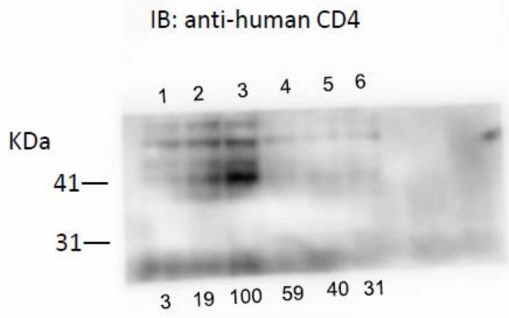

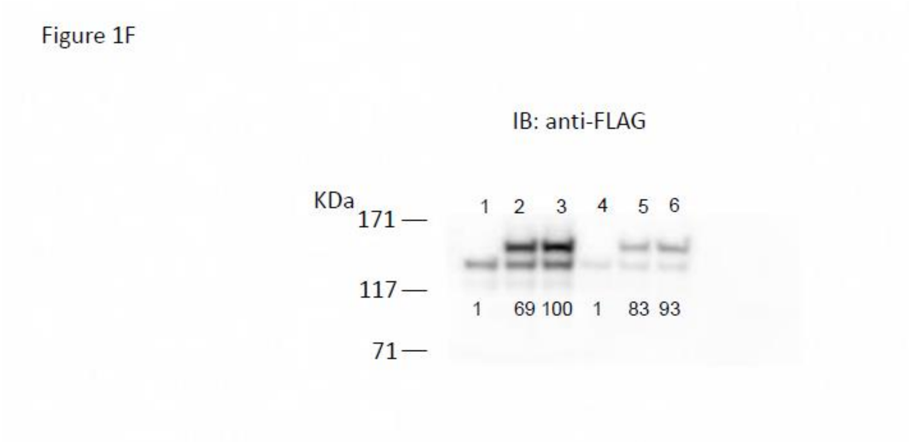

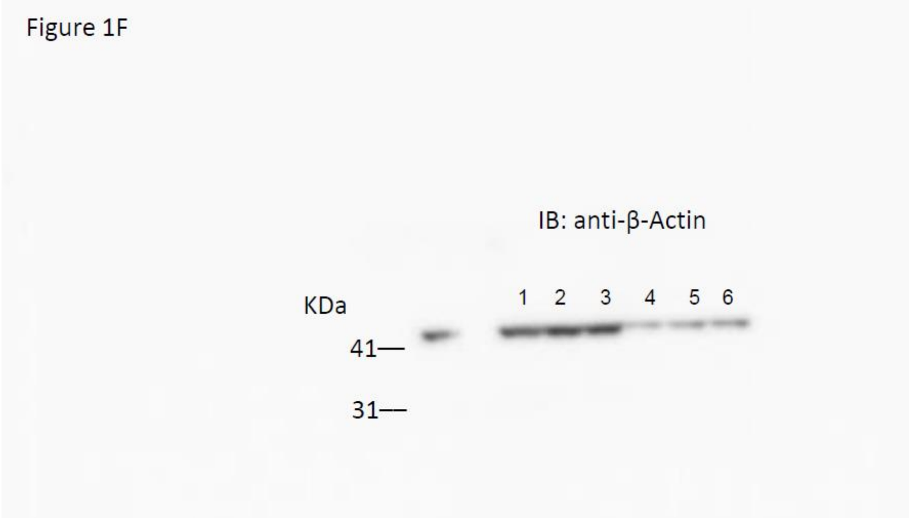

Detail information about Figure 1.

Figure 2A

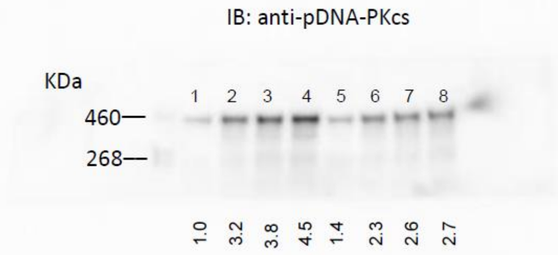

Figure 2A

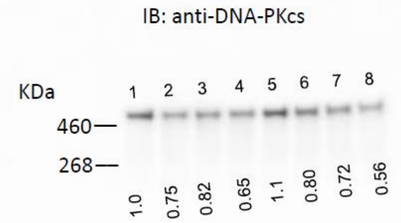

Figure 2A

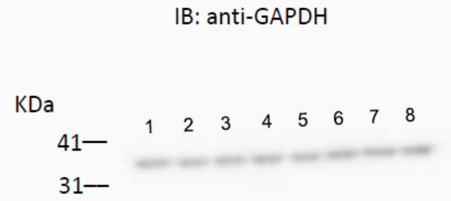

Figure 2C

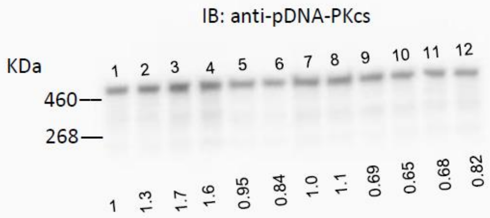

Figure 2C

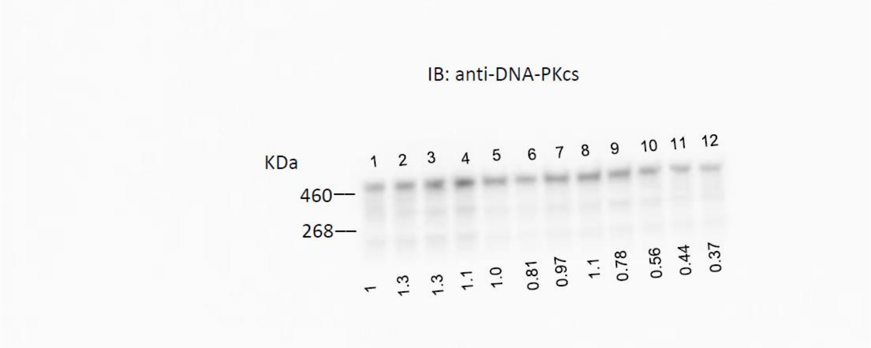

Figure 2C

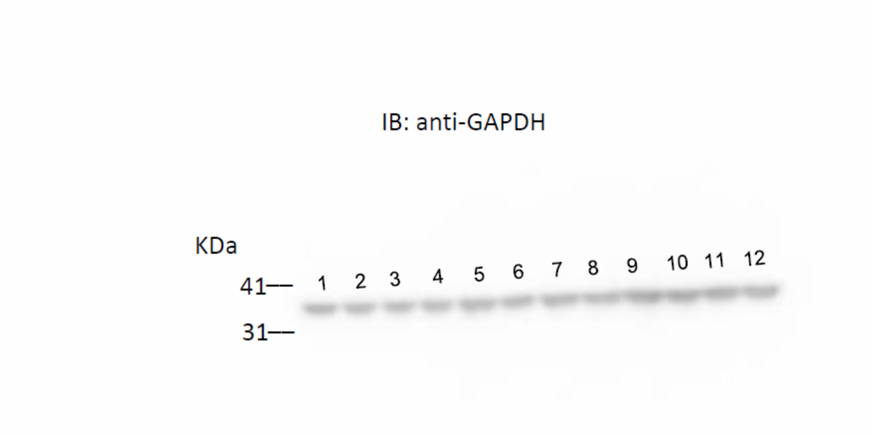

Detail information about Figure 2.

Figure 5A

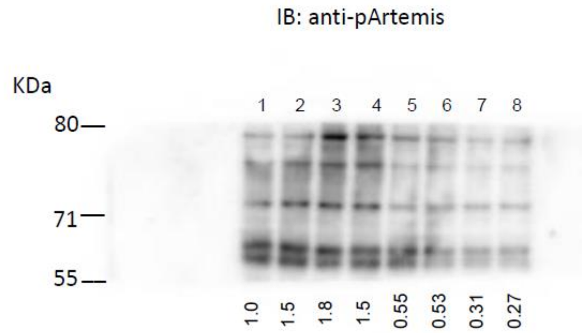

Figure 5A

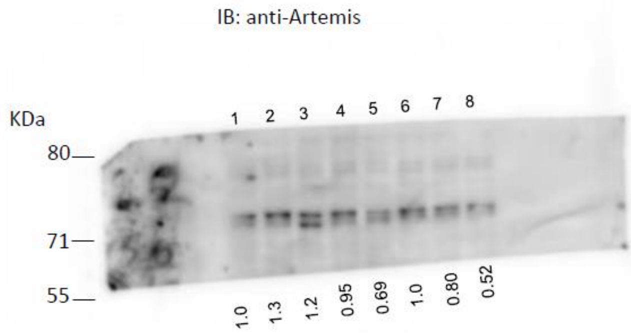

Figure 5C

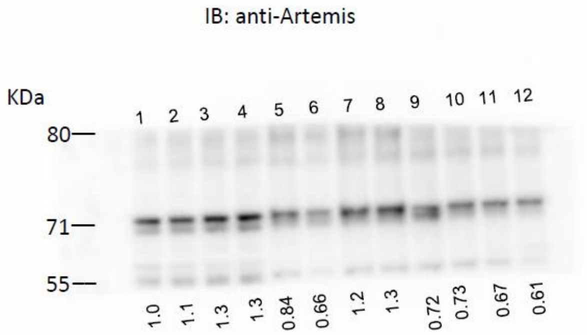

Figure 5C

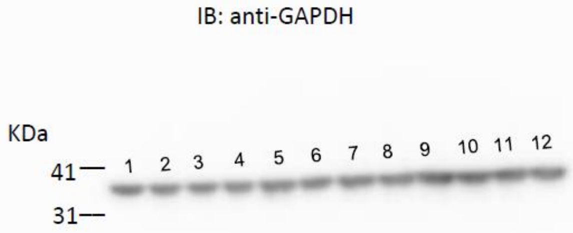

Detail information about Figure 5.

Figure 6A

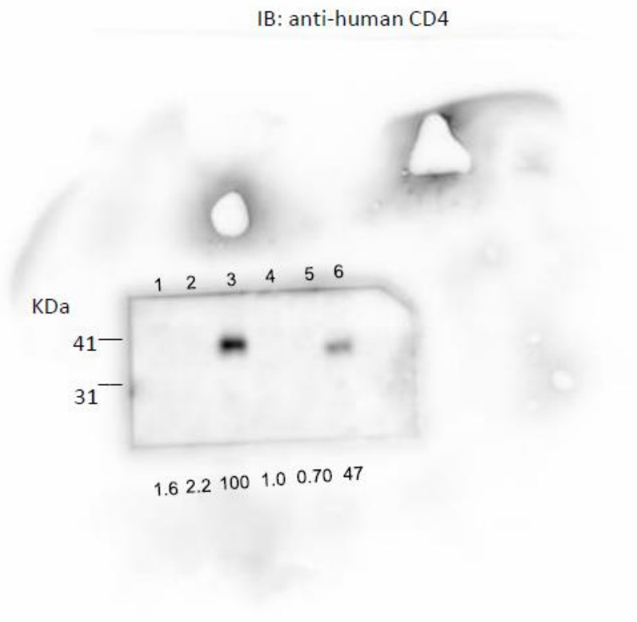

Figure 6A

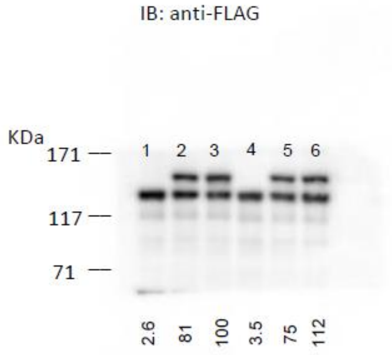

Figure 6A

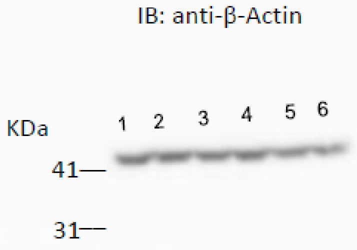

Figure 6C

IB: anti-pDNA-PKcs

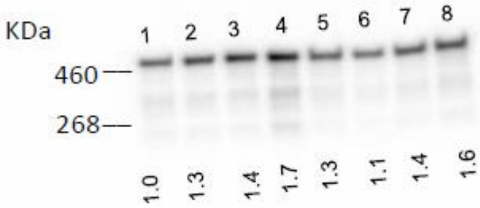

Figure 6C

IB: anti-DNA-PKcs

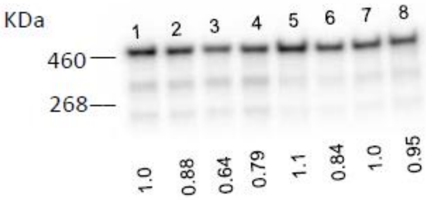

Figure 6C

IB: anti-GAPDH

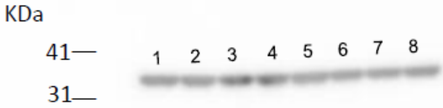

Figure 6G

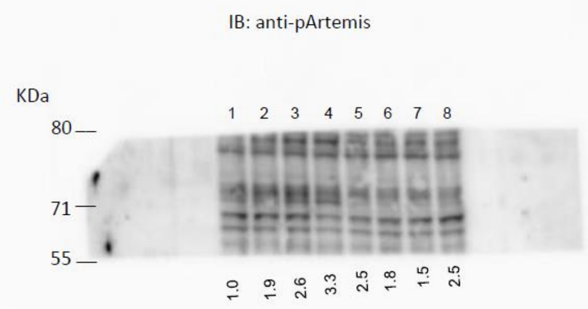

Figure 6G

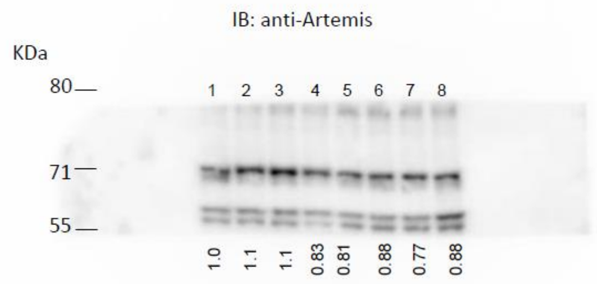

Figure 6G

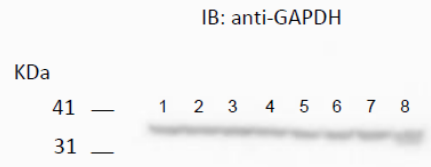

Detail information about Figure 6.

Supplemental figure S2

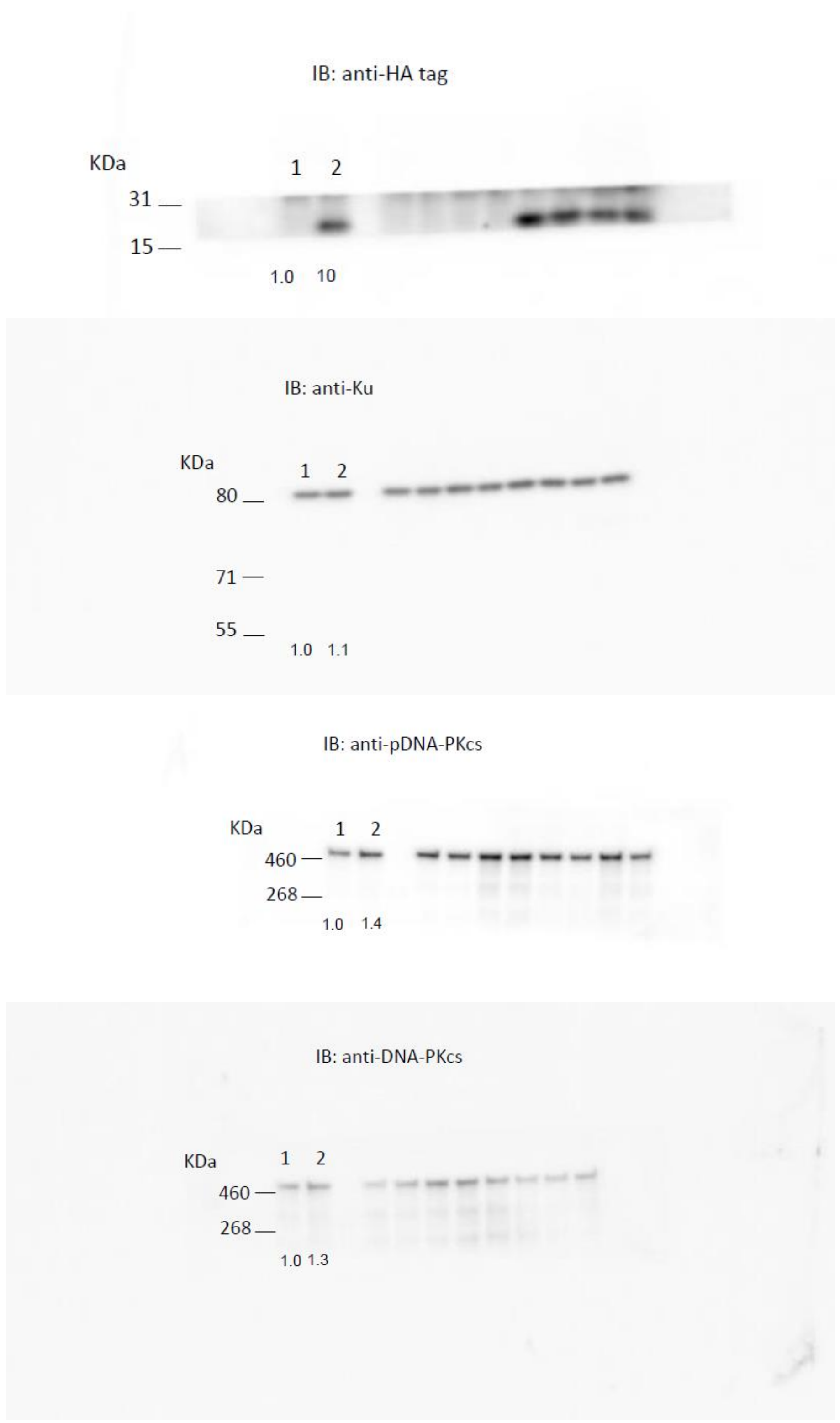

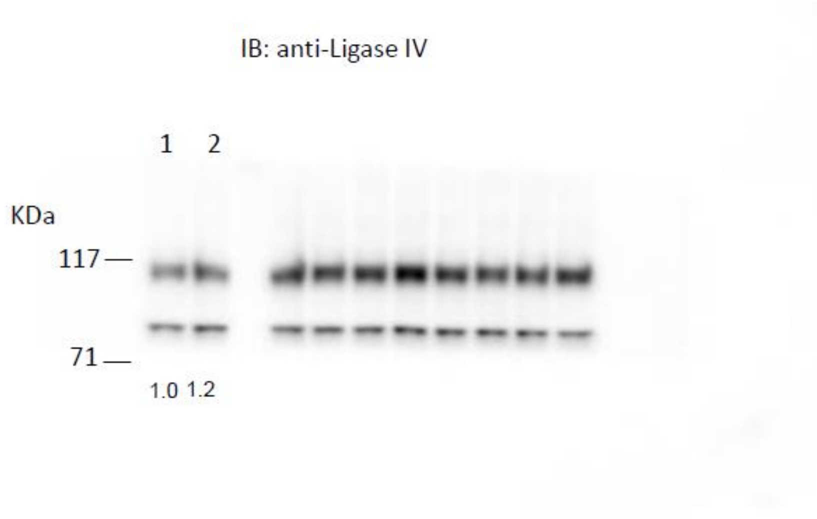
IB: anti-Ligase IV
1
2
KDa
117
71
1.0
1.2

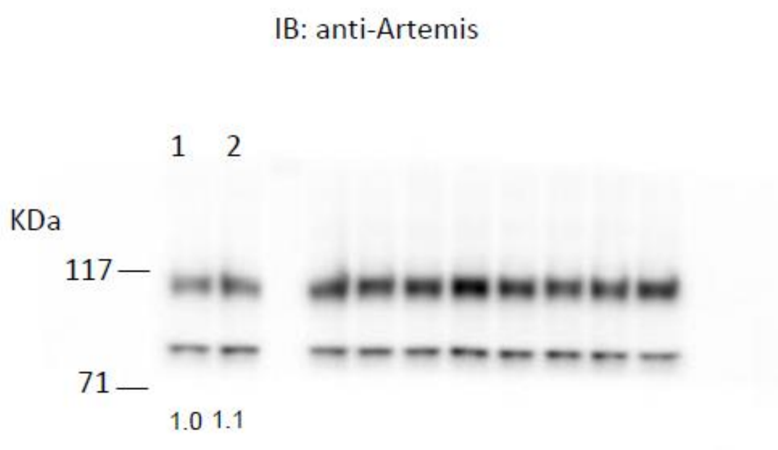
IB: anti-Artemis
1
2
KDa
117
71
1.0
1.1

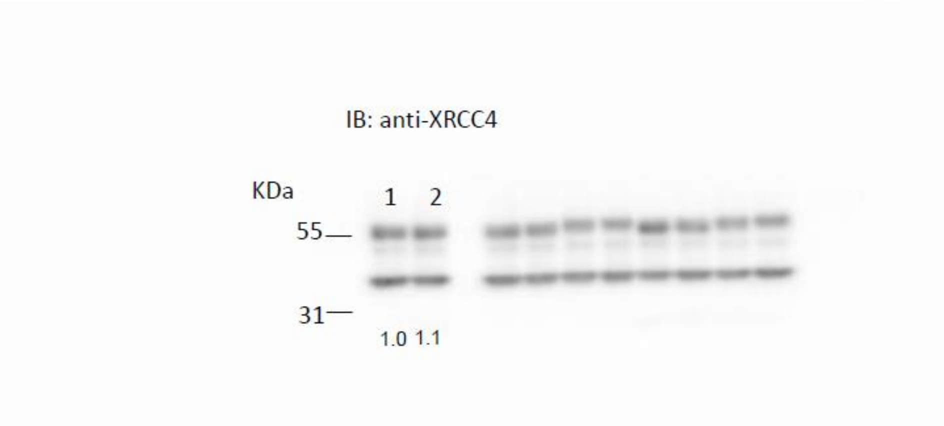
IB: anti-XRCC4
KDa
1
2
55
31
1.0
1.1

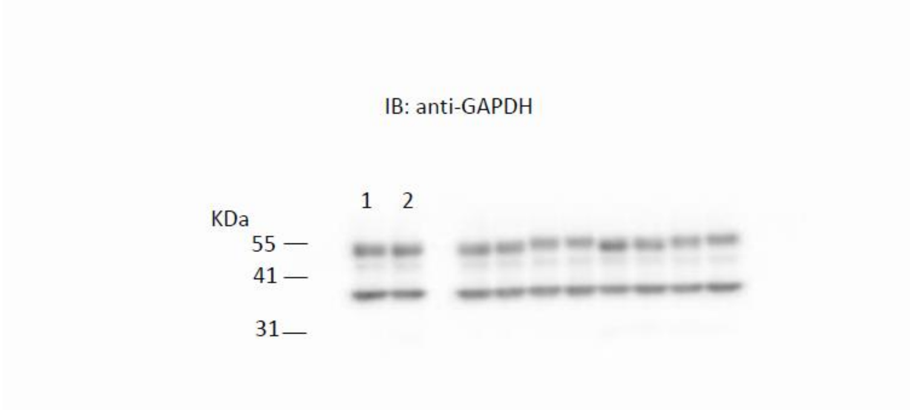
IB: anti-GAPDH
1
2
KDa
55
41
31

Supplemental figure S6

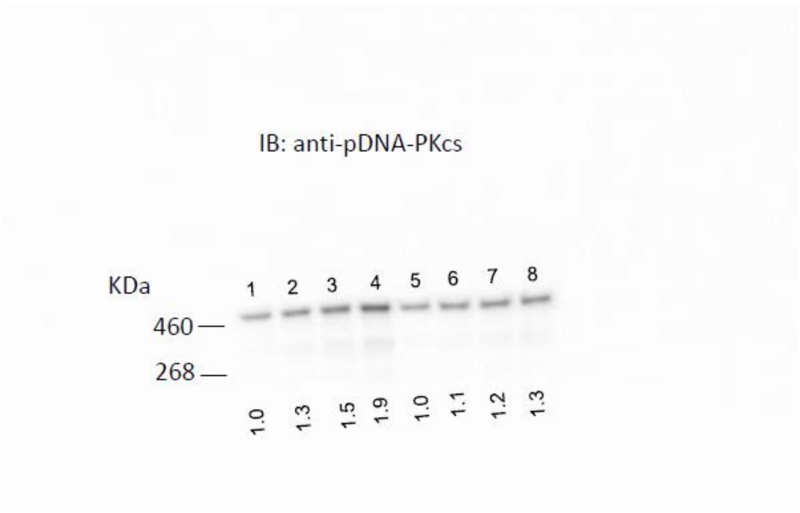

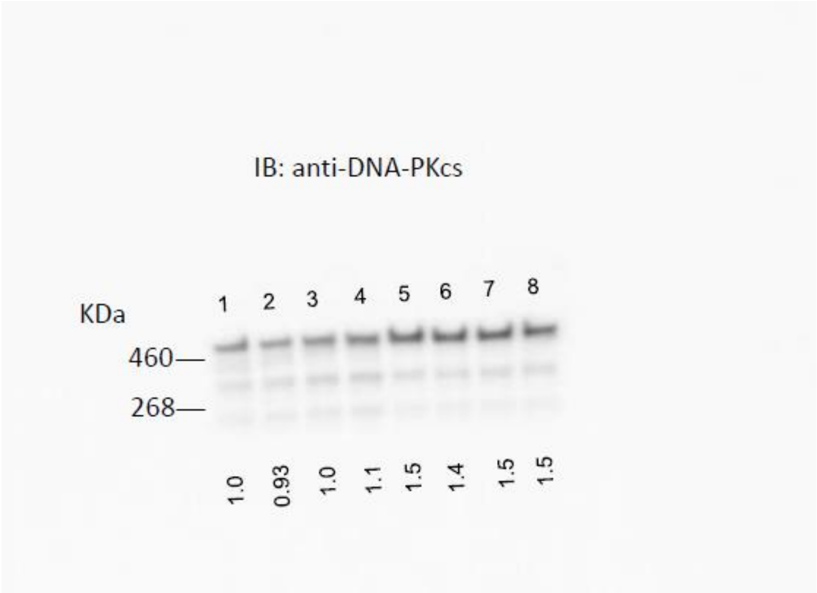

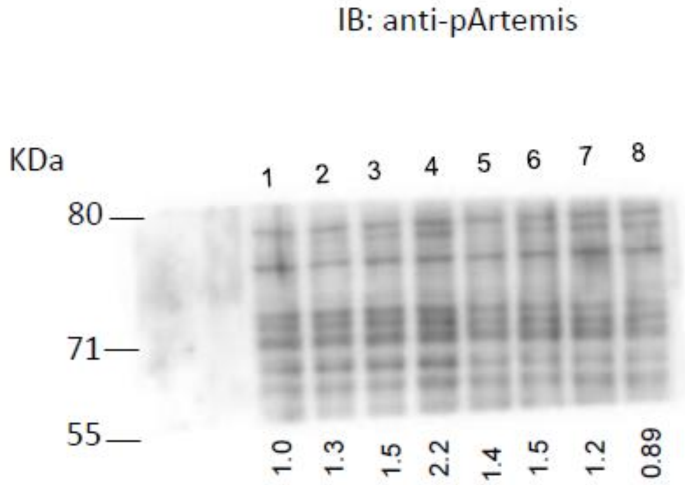

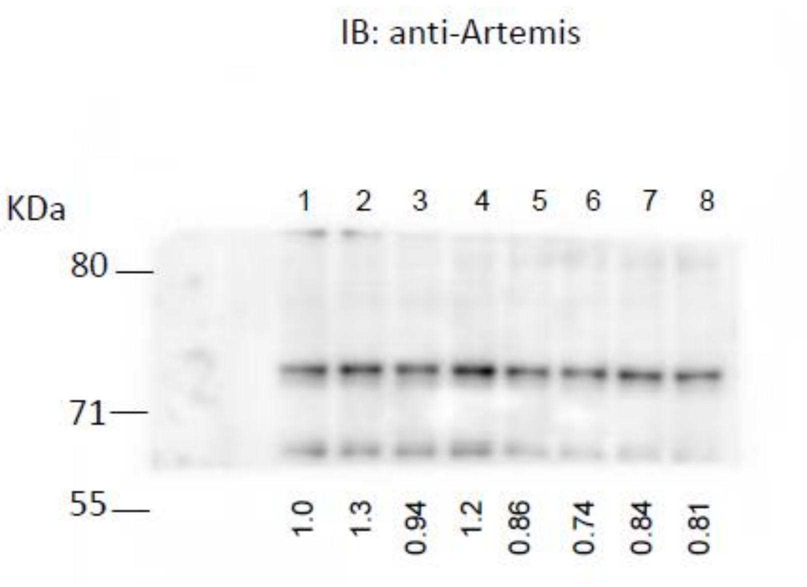
IB: anti-Artemis
KDa
1 2 3 4 5 6 7 8
80
71
55
1.0
1.3
0.94
1.2
0.86
0.74
0.84
0.81

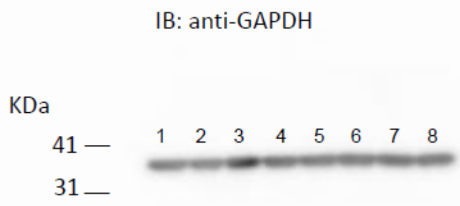
IB: anti-GAPDH
KDa
1 2 3 4 5 6 7 8
41
31

Supplemental figure S8

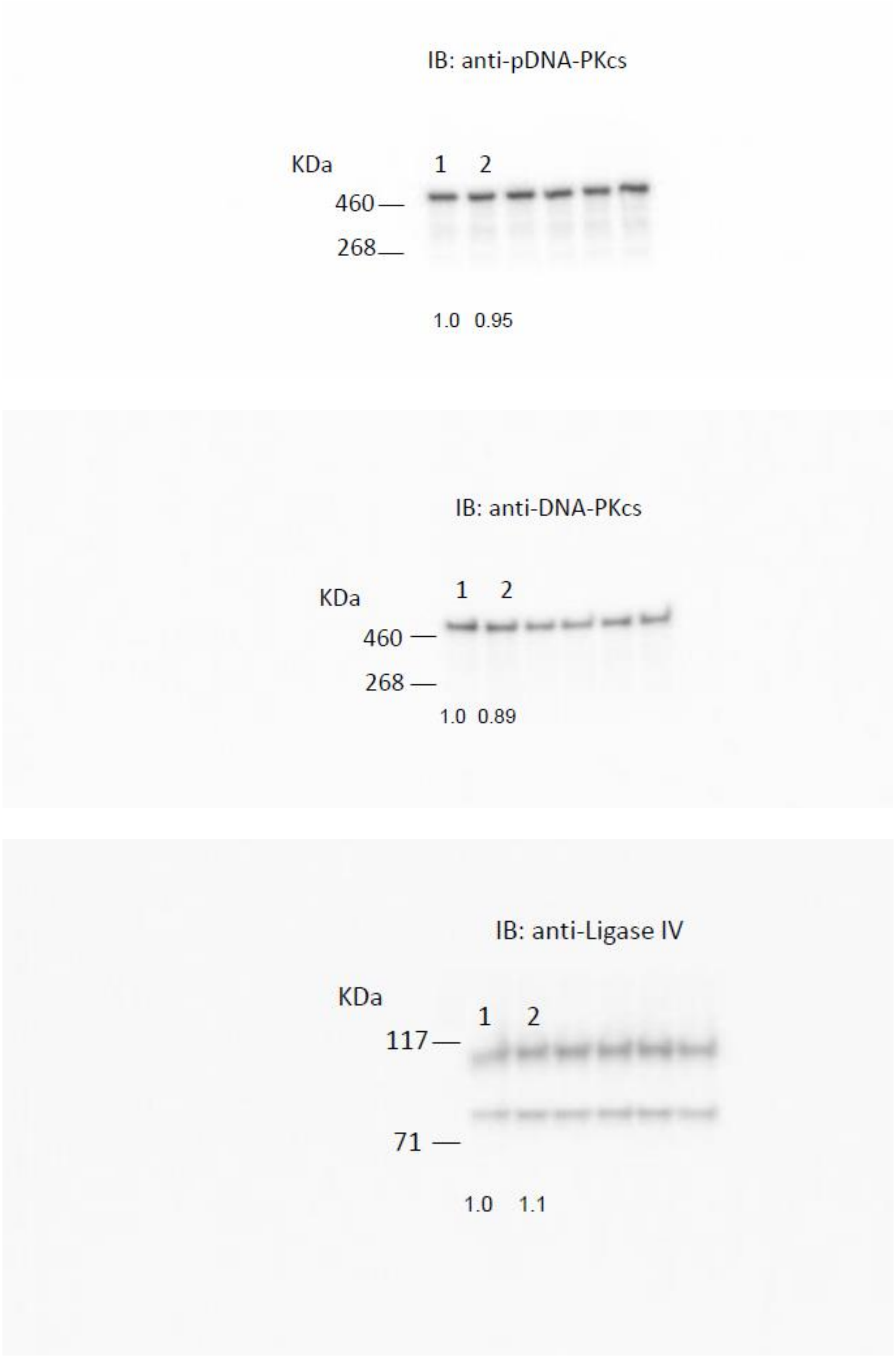

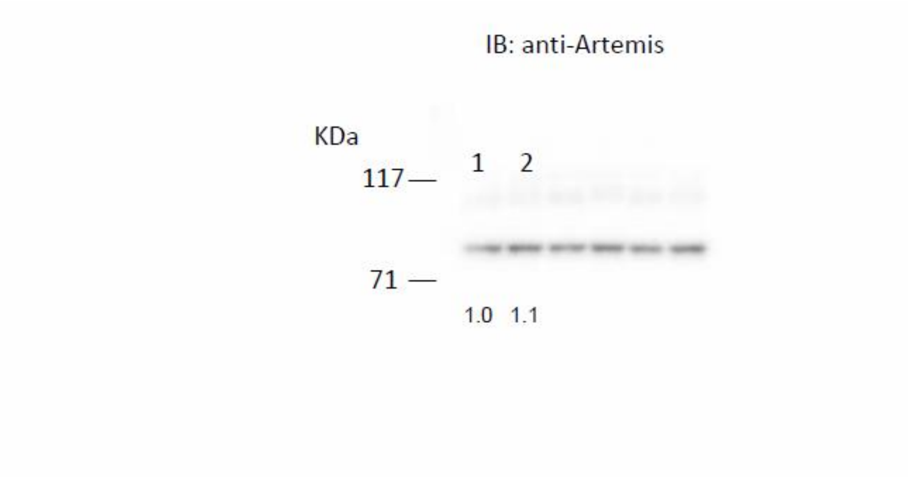

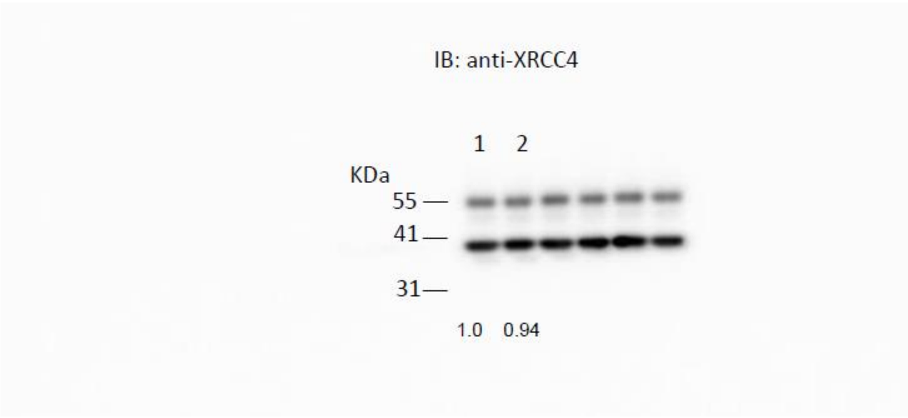

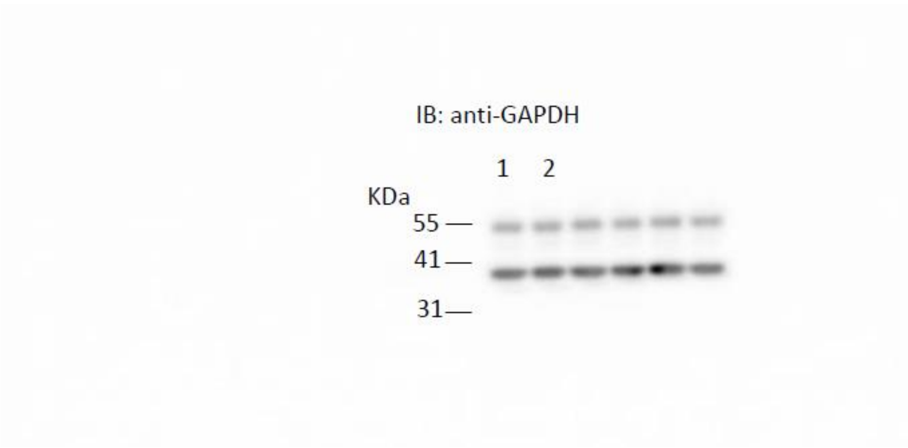

© 2020 by the authors. Licensee MDPI, Basel, Switzerland. This article is an open access article distributed under the terms and conditions of the Creative Commons Attribution (CC BY) license (http://creativecommons.org/licenses/by/4.0/).
